# *NEDD9* overexpression: Prognostic and guidance value in acute myeloid leukaemia

**DOI:** 10.1111/jcmm.16870

**Published:** 2021-08-25

**Authors:** Shenghao Hua, Tao Feng, Lei Yin, Qi Wang, Xuejun Shao

**Affiliations:** ^1^ Department of Clinical Laboratory Children's Hospital of Soochow University Suzhou China

**Keywords:** AML, expression, *NEDD9*, prognosis

## Abstract

It has been demonstrated that neural precursor cell expressed developmentally downregulated protein (*NEDD*) plays crucial roles in tumorigenesis and may serve as potential biomarkers in cancer diagnosis and prognosis. However, few studies systematically investigated the expression of *NEDD* family members in acute myeloid leukaemia (AML). We systemically determined the expression of *NEDD* family members in AML and determined their clinical significance. We identified that *NEDD9* expression was the only member among *NEDD* family which was significantly increased in AML. *NEDD9* overexpression was more frequently classified as FAB‐M4/M5 (*p *= 0.008 and 0.013, respectively), hardly as FAB‐M2/M3. Moreover, *NEDD9* overexpression was significantly associated with complex karyotype and *TP53* mutation. The significant association between *NEDD9* overexpression and survival was also observed in whole‐cohort AML and non‐M3 AML patients. Notably, AML patients with *NEDD9* overexpression may benefit from hematopoietic stem cell transplantation (HSCT), whereas those cases without *NEDD9* overexpression did not. Finally, a total of 822 mRNAs and 31 microRNAs were found to be differentially expressed between two groups. Among the microRNAs, *miR*‐*381* was also identified as a microRNA that could direct target *NEDD9*. Taken together, our findings demonstrated that *NEDD9* overexpression is associated with genetic abnormalities as well as prognosis and might act as a potential biomarker guiding the choice between HSCT and chemotherapy in patients with AML after achieving complete remission.

## 1 INTRODUCTION

Acute myeloid leukaemia (AML) is a blood cancer characterized by clonal myeloid precursors in bone marrow (BM), leading to haematopoiesis failure.^1^ Clinical outcome of AML is highly heterogeneous, survive time from days to cure.^1^ Cytogenetic abnormalities and gene mutations obtained at the diagnosis time provide the most important information.^2^ Recently, aberrant gene expression has also been found to be associated with prognosis in AML, such as *BAALC*, *MN1*, *ERG*, and *WT1*.^3^ Therefore, identification of newly developed biomarkers and construction of molecular‐based prognostic risk scores could more precisely recognize the patients who are at high risk, and finally give intensive treatment to improve their clinical outcome.

*NEDD* (neural precursor cell expressed developmentally downregulated protein) family members (*NEDD1*/*NEDD4*/*NEDD8*/*NEDD9*, *NEDD1*/*4*/*8*/*9*) function highly heterogeneous during biological progress.^4^ *NEDD1*/*4*/*8* members are seen as E3 ubiquitin ligase that recognizes substrates through protein‐protein interactions.^4^, ^5^, ^6^ *NEDD9* is initially identified by its developmentally regulated expression pattern in the early embryonic, but not adult, mouse brain.^7^, ^8^ Dysregulation of *NEDD* family members has been reported in diverse human cancers. For instance, Fujita et al revealed that *NEDD1* expression silencing by siRNA might provide a new opportunity in the treatment of the peritoneal metastasis of scirrhous gastric cancer.^9^ *NEDD4* is widely studied and mostly functions as an oncogene in human cancers, such as gastric cancer, colorectal cancer, lung adenocarcinoma, non‐small‐cell lung carcinoma, hepatocellular carcinoma, breast cancer, and endometrial cancer.^5^, ^10^, ^11^ Oncogenic role of *NEDD8* has been demonstrated in diverse human cancers.^6^, ^12^, ^13^, ^14^ Notably, NEDD8‐activating enzyme inhibitor MLN4924 (pevonedistat) has been used in clinical treatment of AML.^6^, ^15^, ^16^, ^17^ *NEDD9*, as a member of the CAS family of adhesion docking proteins, plays a key role in regulating several signalling cascades related to multiple activities, including migration, adhesion, cell death or proliferation.^18^, ^19^ Overexpression of *NEDD9* has now been strongly linked to poor prognosis in various types of cancers, as well as resistance to first‐line chemotherapeutics.^20^ However, *NEDD9* exhibits opposite effects regarding migratory capacity on myeloid cells as compared to epithelial or lymphoid cells, which block migration and dissemination of neoplastic cells of the myeloid lineage, while stimulating them in solid tumours or lymphoid neoplasias. *NEDD9* −/− mice show an increased number of macrophages, and a simultaneous reduction of B lymphocytes in peripheral blood^21^ and secondary lymphoid organs, yielding an almost complete loss of marginal zone B cells in the spleen.^22^ *NEDD9*‐deficient p210‐BCR/ABL transgenic mice show an increased number of granulocytes in peripheral blood, a hyperplasia of myeloid and megakaryocytic cells in the bone marrow and a diffuse myeloid infiltration in the spleen, lung and liver, leading to earlier progression and shorter mouse survival, which support *NEDD9* capacity to block chronic myeloid leukaemia (CML) progression.^23^ There are few studies on the association of *NEDD9* and AML.^24^

Herein, as far as known, we for the first time identified and verified that *NEDD9* expression, among *NEDD* family members, was significantly increased in AML. *NEDD9* overexpression was correlated with specific cytogenetic and genetic abnormalities of AML. Moreover, *NEDD9* overexpression predicts poor clinical outcome in AML and might act as a potential biomarker guiding treatment selection between chemotherapy and hematopoietic stem cell transplantation (HSCT) as consolidation therapy.

## 2 MATERIALS AND METHODS

### 2.1 GEPIA analysis

The Gene Expression Profiling Interactive Analysis (GEPIA) database (http://gepia.cancer‐pku.cn/) provides RNA‐sequencing expression data of 9,736 tumours and 8587 normal samples from The Cancer Genome Atlas (TCGA) and the Genotype‐Tissue Expression (GTEx) projects, using a standard processing pipeline.^25^ The expression of *NEDD* family members between AML and control was identified by GEPIA.

### 2.2 TCGA data

A total of 173 AML patients with RNA‐sequencing data from the databases of TCGA were included in this study.^26^ *NEDD* family member expression data of these patients were obtained by mRNA sequencing. Mutation data of these patients were also obtained by DNA sequencing. Clinical characteristics and treatment regimens of these patients were also obtained.

### 2.3 Bioinformatic analysis

To obtain the differential expressed genes (DEGs), analysis of RNA‐sequencing (mRNA and microRNA) data was calculated using the raw read counts with the R/Bioconductor package ‘edgeR’. All analyses were controlled for the false discovery rate (FDR) by the Benjamini‐Hochberg procedure. Functional and signalling pathway enrichments were analysed through the STRING (http://string‐db.org). *NEDD9* targeted by microRNA was identified by DIANA (http://diana.imis.athena‐innovation.gr/DianaTools/index.php?r=microT_CDS/index), miRDB (http://mirdb.org/miRDB/), TargetScan (http://www.targetscan.org/vert_72/) and starBase (http://starbase.sysu.edu.cn/).

### 2.4 Statistical analysis

Student t/Mann–Whitney *U*/Kruskal–Wallis *H* test and Pearson's χ^2^/Fisher's exact test were applied for the comparison of continuous and categorical variables, respectively. The effect of *NEDD* family member expression on overall survival (OS) and leukaemia‐free survival (LFS) was analysed by the Kaplan‐Meier method (log‐rank test). Two‐sided *p*‐values less than 0.05 in all statistical analyses were considered as statistically significant differences.

## 3 RESULTS

### 3.1 Expression of NEDD family in AML

**FIGURE 1:**
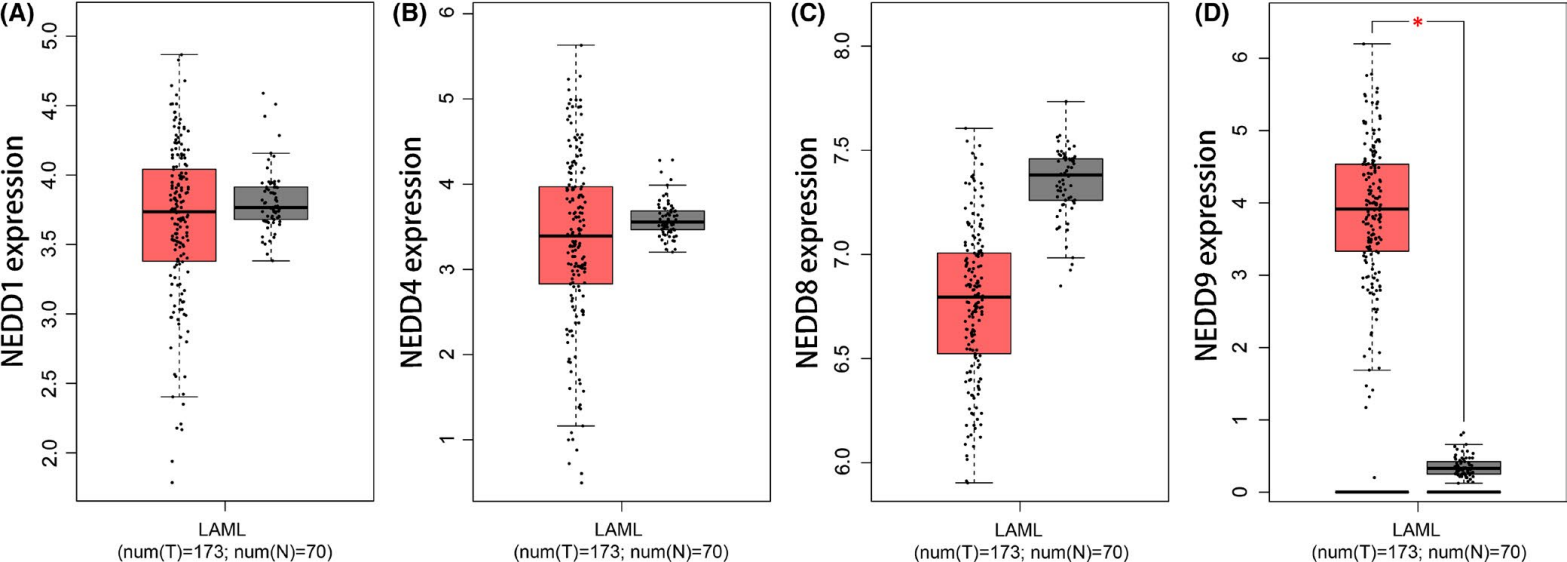
*NEDD* expression in AML. (A): *NEDD1* expression in AML from TCGA data sets using the GEPIA. (B): *NEDD4* expression in AML from TCGA data sets using the GEPIA. (C): *NEDD8* expression in AML from TCGA data sets using the GEPIA. (D): *NEDD9* expression in AML from TCGA data sets using the GEPIA. The red column indicates AML, whereas the grey column indicates control

In order to investigate the *NEDD* family (*NEDD1*/*4*/*8*/*9*) expression patterns in AML, we first used the AML cohort from public databases by GEPIA online website. The AML patients were from the TCGA data sets, whereas normal controls were from the GTEx projects. The expression of *NEDD1*/*4*/*8* showed no significant differences between AML and controls (Figure 1A‐C). However, *NEDD9* expression was markedly increased in AML (Figure 1D).

### 3.2 Clinical implications of NEDD9 expression in AML

**FIGURE 2:**
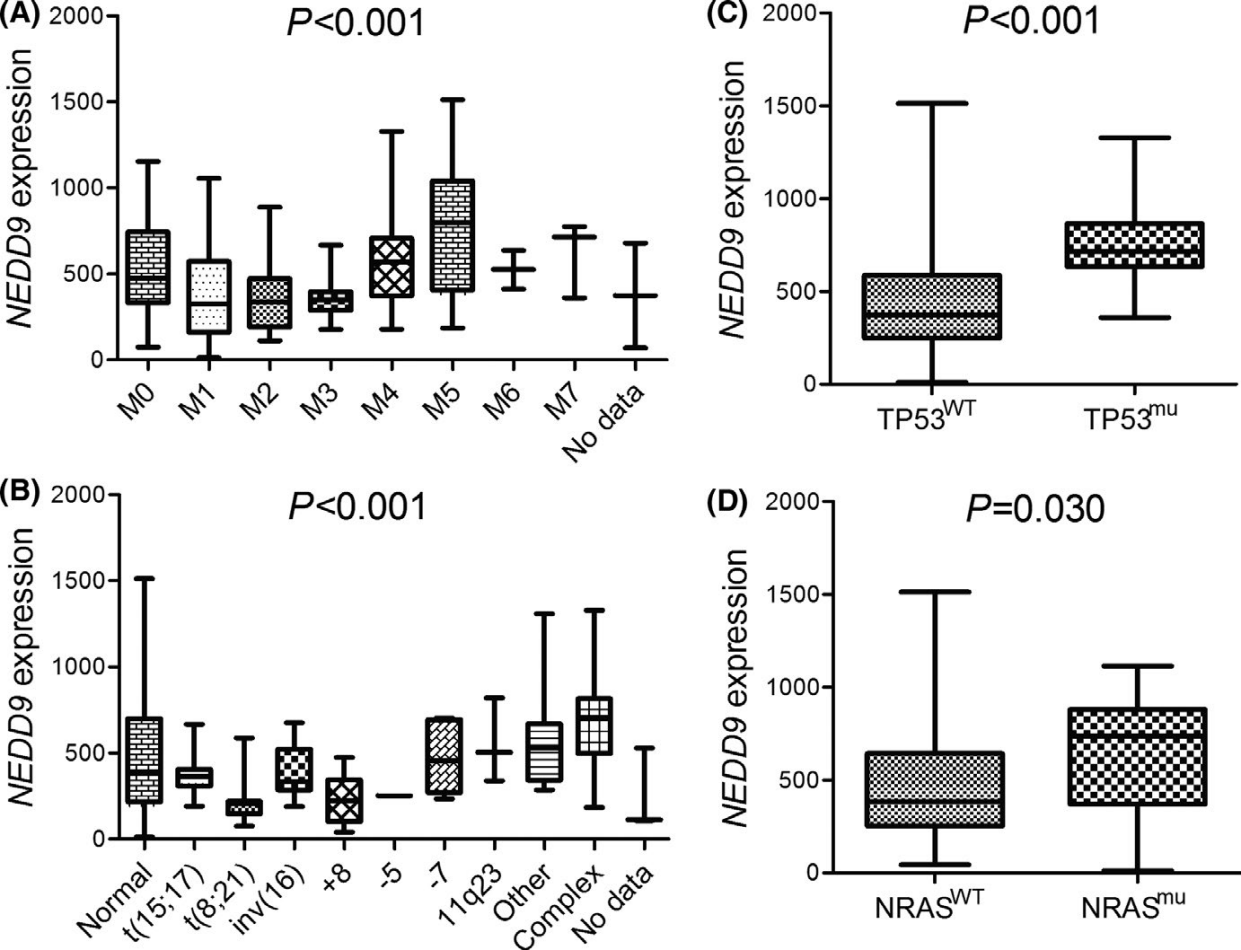
The associations of *NEDD9* expression with FAB classifications and cytogenetic/genetic abnormalities in AML. (A): *NEDD9* expression among FAB subtypes. *p* value indicates the comparison of all FAB subtypes using Kruskal–Wallis *H* test. (B): *NEDD9* expression among different cytogenetics. *p* value indicates the comparison of all cytogenetics using Kruskal–Wallis *H* test. (C): *NEDD9* expression in AML patients with and without *TP53* mutations. (D): *NEDD9* expression in AML patients with and without *NRAS* mutations

Since *NEDD9* was the only one member of *NEDD* family to be aberrantly expressed in AML, we further analysed the correlations of abnormal *NEDD9* expression (above the median level) with the clinical/biological characteristics. As shown in Table 1, AML cases with *NEDD9* overexpression had a markedly older age than those without *NEDD9* overexpression (*p *= 0.003). Interestingly, *NEDD9* overexpressed patients had significantly lower peripheral blood blasts than *NEDD9* underexpressed patients (*p *< 0.001). Moreover, significant differences in the distribution of FAB (French‐American‐British) classification and cytogenetics were found between *NEDD9* overexpressed and underexpressed groups (both *p *= 0.001). *NEDD9* overexpression was more frequently classified as FAB‐M4/M5 (*p *= 0.008 and 0.013, respectively), hardly as FAB‐M2/M3 (*p *= 0.043 and 0.063, respectively). Notably, the frequency of *NEDD9* overexpression in the monocytic line subtype (M4/M5) (38/52, 73.1%) was significantly higher than all the other subtypes (48/121, 39.7%) (*p *< 0.001), whereas the frequency of *NEDD9* overexpression in granulocytic line subtype (M0/M1/M2/M3) (43/114, 37.7%) was markedly lower than all the other subtypes (43/59, 72.9%) (*p *< 0.001). Moreover, *NEDD9* overexpression was significantly associated with complex karyotype (*p *< 0.001). Moreover, *NEDD9* expression pattern was further compared among different FAB subtypes and karyotypes (Figure 2A,B). Among gene mutations, *NEDD9* overexpression was markedly correlated with *TP53* mutation (*p *= 0.001). Additionally, *NEDD9* expression was further compared between the mutant and wild‐type groups of these genes (*TP53* and *NRAS*) (Figure 2C,D).

**TABLE 1:** Correlation of *NEDD9* expression with clinic‐pathologic characteristics in AML

Patient's parameters	*NEDD9* expression		
	Low (*n* = 87)	High (*n* = 86)	*p* Value
Sex, male/female	42/45	50/36	0.224
Median age, years (range)	55 (18–82)	62 (23–88)	0.003
Median WBC, ×10^9^/L (range)	17.9 (0.4–297.4)	15.6 (0.7–137.2)	0.189
Median PB blasts, % (range)	49 (0–98)	17 (0–90)	0.000
Median BM blasts, % (range)	75 (33–100)	71 (30–97)	0.099
FAB classifications			0.001
M0	7	9	
M1	27	17	
M2	25	13	
M3	12	4	
M4	10	24	
M5	4	14	
M6	0	2	
M7	1	2	
No data	1	1	
Cytogenetics			0.001
Normal	41	39	
t(15;17)	11	4	
t(8;21)	6	1	
inv(16)	7	3	
+8	7	1	
del(5)	1	0	
−7/del(7)	3	4	
11q23	1	2	
Others	4	10	
Complex	4	21	
No data	2	1	
Gene mutation			
FLT3 (+/‐)	27/60	22/64	0.500
NPM1 (+/‐)	25/62	23/63	0.865
DNMT3A (+/‐)	20/67	22/64	0.725
IDH2 (+/‐)	7/80	10/76	0.456
IDH1 (+/‐)	10/77	6/80	0.423
TET2 (+/‐)	8/79	7/79	1.000
RUNX1 (+/‐)	6/81	9/77	0.407
TP53 (+/‐)	1/86	13/73	0.001
NRAS (+/‐)	3/84	9/77	0.080
CEBPA (+/‐)	8/79	5/81	0.566
WT1 (+/‐)	7/80	3/83	0.329
PTPN11 (+/‐)	4/83	4/82	1.000
KIT (+/‐)	4/83	3/83	1.000
U2AF1 (+/‐)	4/83	3/83	1.000
KRAS (+/‐)	3/84	4/82	0.720

Abbreviations: AML, acute myeloid leukaemia; BM, bone marrow; FAB, French‐American‐British; NS, no significance; PB, peripheral blood; WBC, white blood cells.

### 3.3 Prognostic value of NEDD9 expression in AML

**FIGURE 3:**
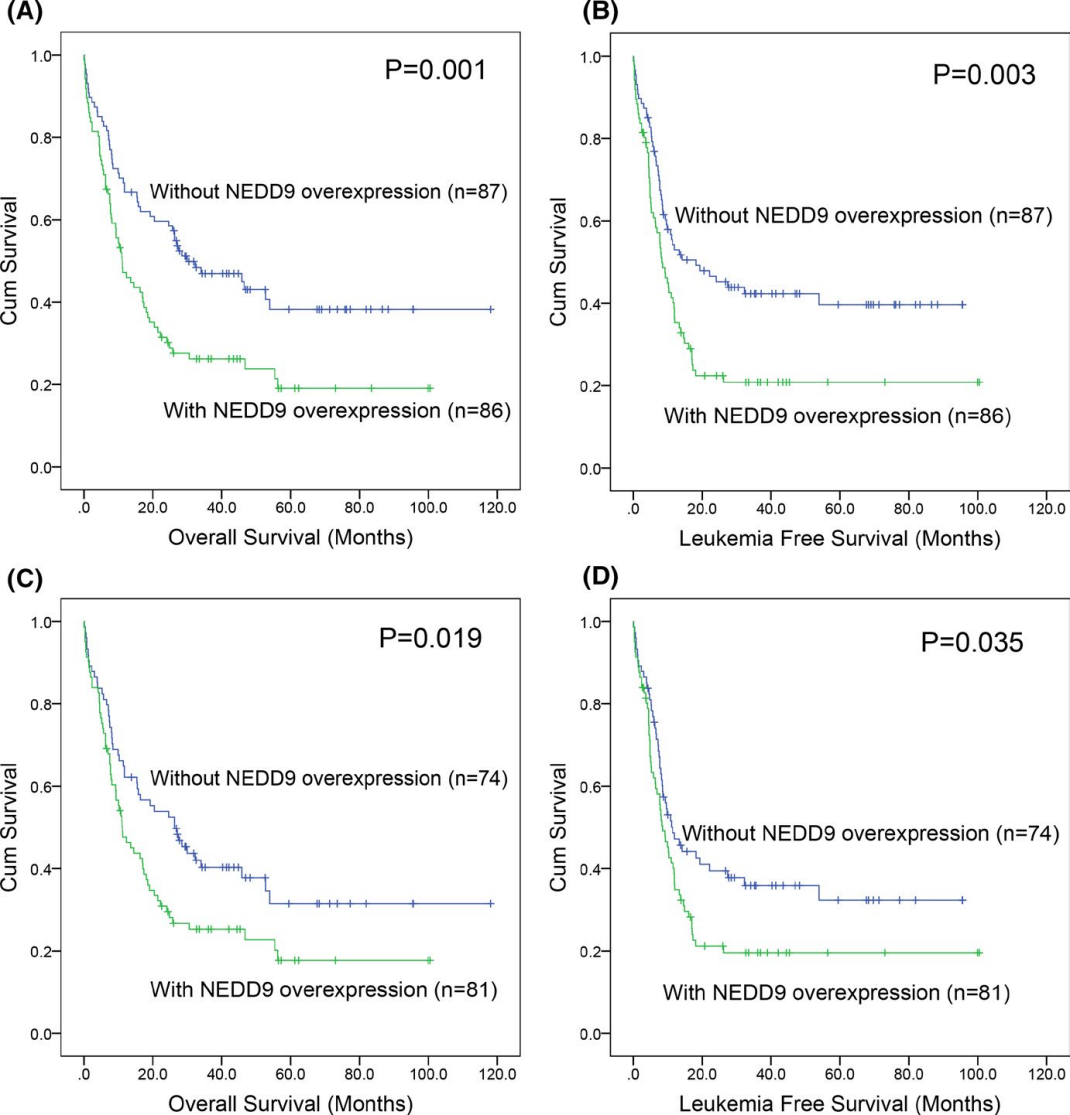
The impact of *NEDD9* expression on survival of AML patients. (A): Kaplan‐Meier survival curves of overall survival in whole‐cohort AML. (B): Kaplan‐Meier survival curves of leukaemia‐free survival in whole‐cohort AML. (C): Kaplan‐Meier survival curves of overall survival in non‐M3 AML. (D): Kaplan‐Meier survival curves of leukaemia‐free survival in non‐M3 AML

To explore the prognostic value of *NEDD9* expression in AML, Kaplan‐Meier analysis was performed and revealed that AML patients with *NEDD9* overexpression presented significantly shorter OS and LFS time than those without *NEDD9* overexpression (Figure 3). Moreover, if FAB‐M3/t(15;17) patients were excluded, non‐M3 AML cases with *NEDD9* overexpression still showed markedly shorter OS and LFS time than those without *NEDD9* overexpression (Figure 3). In addition, we further analysed the prognostic value of the other *NEDD* family (*NEDD1*/*4*/*8*) expression in AML. However, significantly prognostic effect of *NEDD1*/*4*/*8* expression was not identified in AML.

### 3.4 Guidance value of NEDD9 expression in AML

**FIGURE 4:**
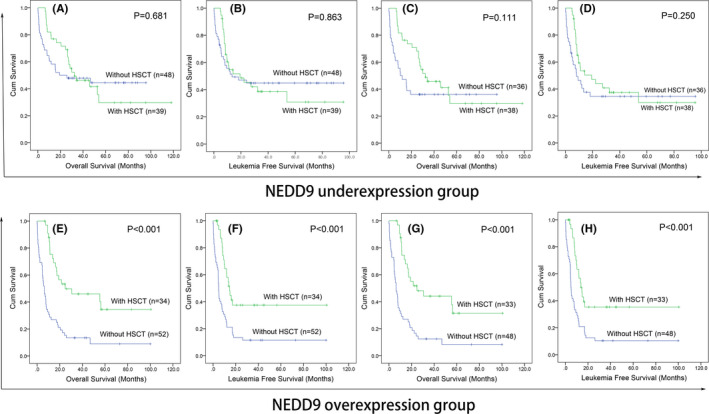
The effect of HSCT on survival of AML patients among *NEDD9* overexpression and underexpression groups. (A): Kaplan‐Meier survival curves of overall survival among whole‐cohort AML in *NEDD9* underexpression group. (B): Kaplan–Meier survival curves of leukaemia‐free survival among whole‐cohort AML in *NEDD9* underexpression group. (C): Kaplan‐Meier survival curves of overall survival among non‐M3 AML in *NEDD9* underexpression group. (D): Kaplan‐Meier survival curves of leukaemia‐free survival among non‐M3 AML in *NEDD9* underexpression group. (E): Kaplan‐Meier survival curves of overall survival among whole‐cohort AML in *NEDD9* overexpression group. (F): Kaplan–Meier survival curves of leukaemia‐free survival among whole‐cohort AML in *NEDD9* overexpression group. (G): Kaplan‐Meier survival curves of overall survival among non‐M3 AML in *NEDD9* overexpression group. (H): Kaplan–Meier survival curves of leukaemia‐free survival among non‐M3 AML in *NEDD9* overexpression group

HSCT as a consolidation treatment regimen is of great importance in AML against disease recurrence. To investigate whether HSCT might overcome the adverse prognostic effect caused by *NEDD9* overexpression in AML, we analysed the prognostic impact of HSCT in *NEDD9* overexpressed and underexpressed groups, respectively. After AML patients achieved CR, cases undergoing HSCT exhibited markedly longer OS and LFS compared with that only receiving chemotherapy in *NEDD9* overexpressed group. However, in *NEDD9* underexpressed group, there were no significant differences regarding OS and LFS between HSCT and chemotherapy groups (Figure 4). These results suggested that AML patients with *NEDD9* overexpression may benefit from HSCT, and *NEDD9* expression might act as a potential biomarker guiding treatment selection between HSCT and chemotherapy in patients with AML after achieving CR by induction therapy.

### 3.5 Biological network of NEDD9 expression in AML

**FIGURE 5:**
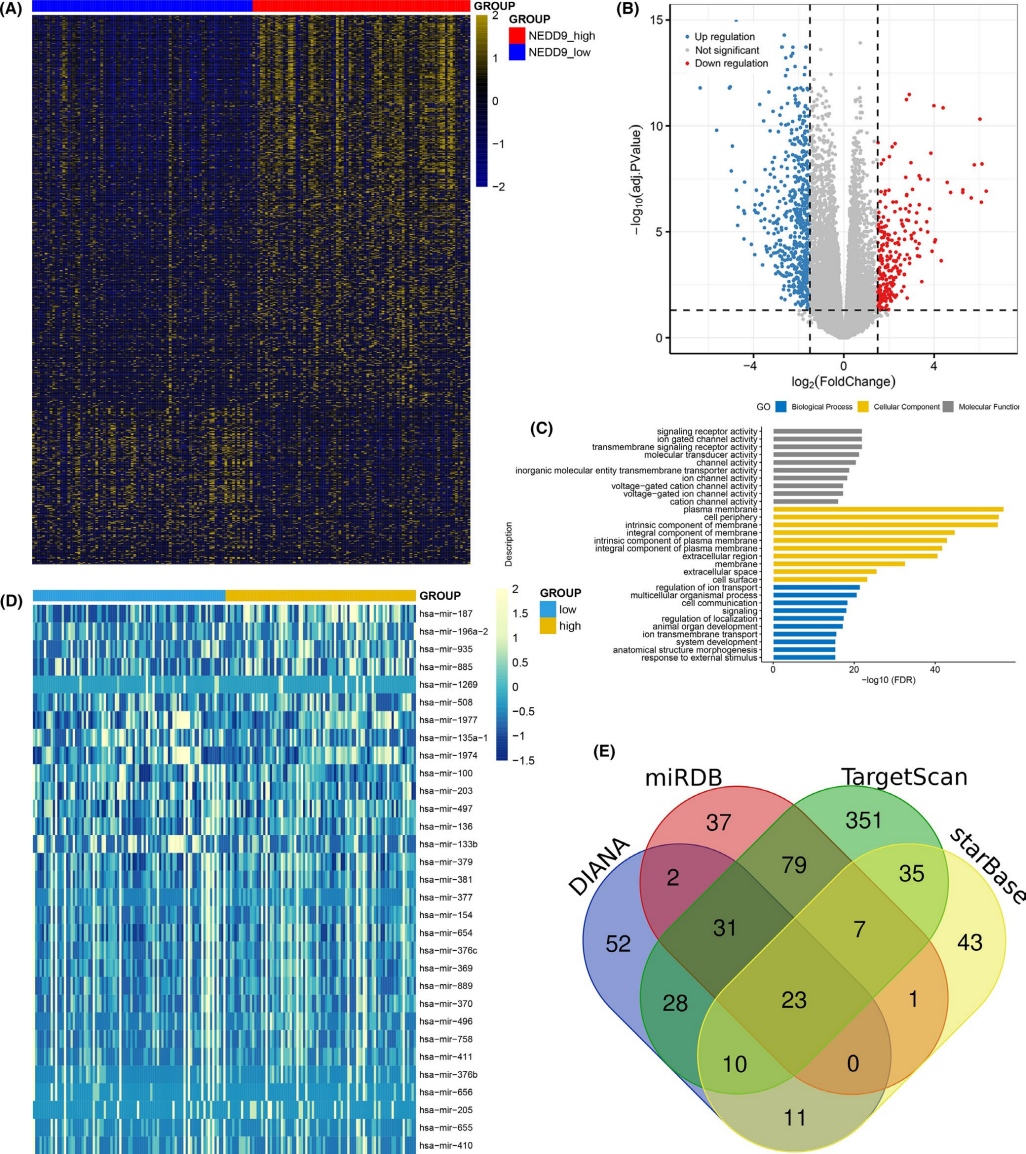
Biological network of *NEDD9* in AML. (A): Expression heatmap of differentially expressed mRNAs between AML patients with *NEDD9* overexpression and underexpression groups. (B): Volcano plot of differentially expressed mRNAs between AML patients with *NEDD9* overexpression and underexpression groups. (C): Gene Ontology analysis of differentially expressed mRNAs conducted using online website of STRING (http://string‐db.org). (D): Expression heatmap of differentially expressed microRNAs between AML patients with *NEDD9* overexpression and underexpression groups. (E): Venn results of microRNAs which could target *NEDD9* predicted by DIANA (http://diana.imis.athena‐innovation.gr/DianaTools/index.php?r=microT_CDS/index), miRDB (http://mirdb.org/miRDB/), TargetScan (http://www.targetscan.org/vert_72/) and starBase (http://starbase.sysu.edu.cn/)

To get better understanding of the biological network associated with *NEDD9* expression in AML, we first compared the transcriptomes of *NEDD9* overexpression and underexpression groups in AML among TCGA cohorts. Based on the filter condition: |log2 FC|>1.5, FDR < 0.05 and *p *<0.05, a total of 822 genes including 588 upregulated and 234 downregulated (high vs low) were found to be differentially expressed between two groups (Figure 5A,B and Table S1). The top 10 upregulated genes such as *FEZ1* and *PDK4* are reported with proto‐leukaemia effects.^27^, ^28^ Furthermore, the Gene Ontology analysis revealed that these genes involved in biologic processes, including multicellular organismal process, cell communication and signalling (Figure 5C).

Moreover, we also compared the microRNA expression pattern between *NEDD9* overexpression and underexpression groups. A total of 31 microRNAs including 6 upregulated and 25 downregulated were found to be differentially expressed between two groups (Figure 5D and Table S1). Downregulated microRNAs such as *miR*‐*135a*, *miR*‐*203*, *miR*‐*497*, *miR*‐*381*, *miR*‐*370* and *miR*‐*758* were found to be underexpressed in AML or have anti‐leukaemia effects in previous reports.^29^, ^30^, ^31^, ^32^, ^33^, ^34^, ^35^ Of these microRNAs, *miR*‐*381* was also identified as a microRNA that could direct target *NEDD9* (Figure 5E and Table S2), which suggested *NEDD9* is a direct target of *miR*‐*381*.

## 4 DISCUSSION

It has been determined that *NEDD9* plays a crucial role in regulating several signalling cascades contained in multiple activities, including cell apoptosis or proliferation, migration, invasion, metastasis and adhesion.^7^ Moreover, overexpression of *NEDD9* correlated with cancer cell development and drug resistance in several types of solid tumours such as lung cancer, melanoma and breast cancer.^8^ It is not surprising that aberrant *NEDD9* expression has been linked to the prognosis of human cancers.^8^, ^20^

In this study, we for the first time revealed that *NEDD9* overexpression, identified from *NEDD* family, was associated with poor prognosis in AML. Notably, *NEDD9* expression might act as a potential biomarker predicting prognosis and guiding treatment choice between chemotherapy and HSCT in AML. Until now, few investigations have reported the links between *NEDD9* and AML. In contrary to our results, Pallarès et al demonstrated that *NEDD9* was an independent good prognostic factor in intermediate‐risk AML patients.^24^ The possible reason was that the previous report only included AML patients less than 65 years. As it is well known, AML is an ageing disease which contains larger numbers of older patients. Accordingly, further clinical and functional studies are needed to evaluate the clinical implication and potential role of *NEDD9* in AML.

Our study also found significant associations between *NEDD9* expression and FAB classifications as well as cytogenetic/genetic subtypes in AML. For FAB classifications, *NEDD9* overexpression was associated with FAB‐M4/M5 in accordance with previous studies,^24^ and results analysed by BloodSopt (https://servers.binf.ku.dk/bloodspot/) show that *NEDD9* expression is significantly higher in monocytic lineages, suggesting it may play a crucial role in monocytic line development (Figure S1). For cytogenetic/genetic subtypes, *NEDD9* overexpression was found to be strongly correlated with complex karyotype and *TP53* mutations. Since *TP53* mutation is frequently occurred in the complex karyotype,^36^ it is difficult to classify which is mainly the factor associated with *NEDD9* overexpression. Interestingly, previous studies have determined the association of *TP53* with *NEDD9* in non‐small‐cell lung cancer.^37^ Moreover, we also confirmed that *TP53* could bind the *NEDD9* promoter with a predicted sequence ACCAGCTCAAACATT by analysing JASPAR (http://jaspar.genereg.net/). These results demonstrated that *NEDD9* overexpression plays a key role in leukaemogenesis caused by complex karyotype and/or *TP53* mutations. Further studies are required to determine the underlying mechanism of *NEDD9* expression in AML with complex karyotype and *TP53* mutations.

*NEDD9* regulated by microRNAs has been reported by several studies. *MiR*‐*25*‐*5p* directly targeting *NEDD9* was found in oral squamous cell carcinoma and colorectal cancer.^38^, ^39^ Moreover, *NEDD9* expression regulated by *miR*‐*145* was revealed in lung cancer, pancreatic cancer, renal cell carcinoma, prostate cancer and glioblastoma.^40^, ^41^, ^42^, ^43^, ^44^ Additionally, *NEDD9* expression negatively associated with *miR*‐*125a*/*b* was shown in pancreatic cancer, lung adenocarcinoma and melanoma.^45^, ^46^, ^47^ In pancreatic cancer and hepatocellular carcinoma, *NEDD9* was reported to be regulated by *miR*‐*18a* playing a key role during carcinogenesis.^48^, ^49^ In our study, we for the first time found that *NEDD9* expression was negatively associated with *miR*‐*381* in AML. However, the limitation in our study was that luciferase assay was not performed to verify the direct associations between *miR*‐*381* and *NEDD9*. Therefore, a number of investigations are needed to confirm our results in the future.

Collectively, our findings demonstrated that *NEDD9* overexpression associated with genetic abnormalities as well as prognosis might act as a potential biomarker guiding the choice between HSCT and chemotherapy in patients with AML.

## CONFLICT OF INTEREST

The authors confirm that there are no conflicts of interest.

## AUTHOR CONTRIBUTIONS

**Shenghao Hua:** Conceptualization (equal); Writing‐original draft (equal). **tao feng:** Methodology (supporting); Writing‐review & editing (supporting). **lei yin:** Methodology (supporting); Software (supporting); Writing‐review & editing (supporting). **qi wang:** Methodology (supporting); Writing‐review & editing (supporting). **xuejun shao:** Conceptualization (equal); Investigation (equal); Project administration (equal); Resources (equal).

## Supporting information

Figure S1

Table S1

Table S2
