# Supplementary figures and images for: NEDD9 overexpression: Prognostic and guidance value in acute myeloid leukaemia
Source: J Cell Mol Med. 2021 Aug 25;25(19):9331–9. doi: 10.1111/jcmm.16870 (PMC8500976; doi:10.1111/jcmm.16870)

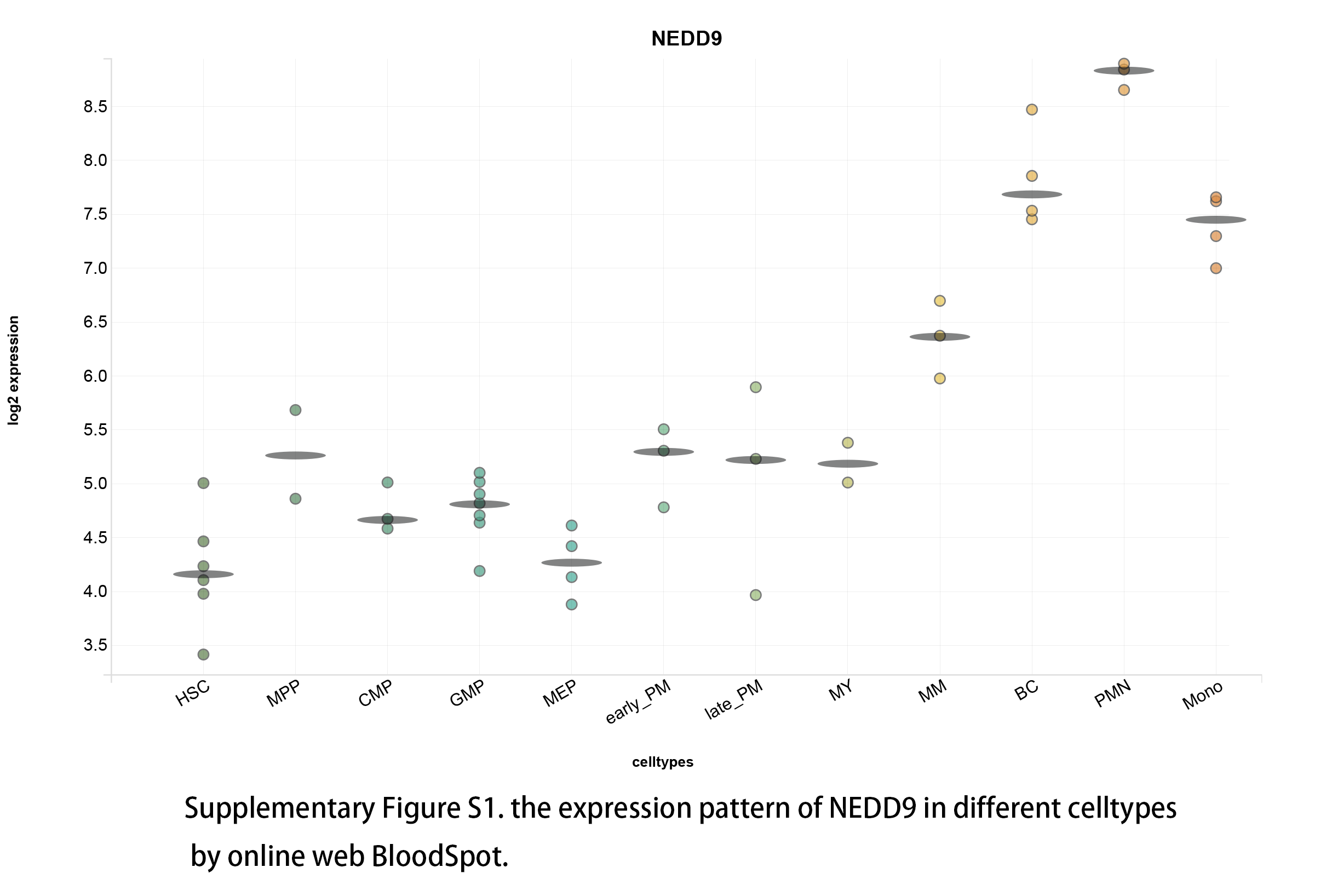

Supplement: Supplementary file 1 — Figure S1 [file JCMM-25-9331-s001.tif]
